# Genetic parameters for novel climatic resilience indicators derived from automatically-recorded vaginal temperature in lactating sows under heat stress conditions

**DOI:** 10.1186/s12711-024-00908-4

**Published:** 2024-06-10

**Authors:** Hui Wen, Jay S. Johnson, Leonardo S. Gloria, Andre C. Araujo, Jacob M. Maskal, Sharlene Olivette Hartman, Felipe E. de Carvalho, Artur Oliveira Rocha, Yijian Huang, Francesco Tiezzi, Christian Maltecca, Allan P. Schinckel, Luiz F. Brito

**Affiliations:** 1 Department of Animal Sciences, Purdue University, West Lafayette, IN USA; 2 USDA-ARS Livestock Behavior Research Unit, West Lafayette, IN USA; 3 Smithfield Premium Genetics, Rose Hill, NC USA; 4 Department of Animal Science, North Carolina State University, Raleigh, NC USA; 5 Department of Agriculture, Food, Environment and Forestry, University of Florence, Florence, Italy

## Abstract

**Background:**

Longitudinal records of automatically-recorded vaginal temperature (T_V_) could be a key source of data for deriving novel indicators of climatic resilience (CR) for breeding more resilient pigs, especially during lactation when sows are at an increased risk of suffering from heat stress (HS). Therefore, we derived 15 CR indicators based on the variability in T_V_ in lactating sows and estimated their genetic parameters. We also investigated their genetic relationship with sows’ key reproductive traits.

**Results:**

The heritability estimates of the CR traits ranged from 0.000 ± 0.000 for slope for decreased rate of T_V_ (Slope_De_) to 0.291 ± 0.047 for sum of T_V_ values below the HS threshold (HSU_B_). Moderate to high genetic correlations (from 0.508 ± 0.056 to 0.998 ± 0.137) and Spearman rank correlations (from 0.431 to 1.000) between genomic estimated breeding values (GEBV) were observed for five CR indicators, i.e. HS duration (HSD), the normalized median multiplied by normalized variance (Nor_medvar), the highest T_V_ value of each measurement day for each individual (Max_Tv_), and the sum of the T_V_ values above (HSU_A_) and below (HSU_B_) the HS threshold. These five CR indicators were lowly to moderately genetically correlated with shoulder skin surface temperature (from 0.139 ± 0.008 to 0.478 ± 0.048) and respiration rate (from 0.079 ± 0.011 to 0.502 ± 0.098). The genetic correlations between these five selected CR indicators and sow reproductive performance traits ranged from − 0.733 to − 0.175 for total number of piglets born alive, from − 0.733 to − 0.175 for total number of piglets born, and from − 0.434 to − 0.169 for number of pigs weaned. The individuals with the highest GEBV (most climate-sensitive) had higher mean skin surface temperature, respiration rate (RR), panting score (PS), and hair density, but had lower mean body condition scores compared to those with the lowest GEBV (most climate-resilient).

**Conclusions:**

Most of the CR indicators evaluated are heritable with substantial additive genetic variance. Five of them, i.e. HSD, Max_Tv_, HSU_A_, HSU_B_, and Nor_medvar share similar underlying genetic mechanisms. In addition, individuals with higher CR indicators are more likely to exhibit better HS-related physiological responses, higher body condition scores, and improved reproductive performance under hot conditions. These findings highlight the potential benefits of genetically selecting more heat-tolerant individuals based on CR indicators.

**Supplementary Information:**

The online version contains supplementary material available at 10.1186/s12711-024-00908-4.

## Background

Heat stress (HS) compromises the health, production, and reproduction performance of animals and causes significant welfare issues in the livestock industry worldwide [1–4]. Numerous strategies for mitigating the adverse effects of HS have been proposed over time, including management changes to promote heat abatement in dairy cattle [5], swine [6], beef cattle [7], and poultry [8]. In addition to improving the environmental conditions in which animals are raised, genetic or genomic selection for improved climate resilience (CR) is a promising route for more sustainable animal production [9]. In this study, CR is defined as the ability of an animal to maintain or rapidly return to euthermia under thermally stressful conditions. More climate-resilient animals can adapt to a wider range of environmental conditions, resulting in less intensive farm management requirements while maintaining good performance and welfare status [10].

Many methods have been proposed to study the genetic background of resilience in livestock. For instance, the slope of reaction norms for animals across environments or different levels of disturbances has been considered as an indicator of animal resilience [11–13]. More recently, many studies have been conducted with a focus on animal resilience. For instance, Sánchez-Molano et al. [14] developed resilience indicators for weather variability and identified more heat-tolerant animals based on daily milk yield data using reaction norm slopes in a UK dairy goat population. Freitas et al. [15] evaluated heat tolerance in Large White pigs based on single-step genomic reaction norms, using routinely-recorded performance variables, and climatic data from public weather stations and reported that heat tolerance is heritable.

Longitudinal records collected at short time intervals can capture the impact of known or unknown disturbances, which can then be used to derive indicators of overall animal resilience [15]. Production performance and physiological states are linked to animal resilience, as evidenced by indicators that are derived from daily milk yield and step count in dairy cattle [16, 17]. Poppe et al. [18] proposed methods (e.g., variance, autocorrelation—the degree of similarity between observations recorded at different points in time [19], and skewness of deviations from lactation curves) to quantify resilience while considering an animals’ overall performance [18]. These methods have been widely used for modelling longitudinally-recorded variables, including body weight [20, 21], daily milk yield [17], and egg production [22].

Measures of body temperature, such as rectal temperature, have been previously used as HS indicators [23, 24]. These measurements, combined with environmental information such as the temperature humidity index (THI), can reflect the ability of an animal to cope with varying climatic conditions [25]. However, measuring these indicators manually can be time-consuming, labor-intensive, and disrupt the normal behavior of animals; thus, automatic recording of the vaginal temperature (T_V_) on a longitudinal scale could be an alternative for assessing diurnal changes in female body temperature [26–28]. In previous studies, we investigated genetic parameters for various HS indicators [29, 30], including body temperatures (skin surface temperature and T_V_), body size, body condition score (BCS), and ear size, and behavioral responses to HS [respiration rate (RR) and panting score (PS)]. Among these traits, T_V_ was the only one that was automatically measured every 10 min, resulting in a substantial number of records. Traits based on T_V_ exhibited moderate heritability estimates at different time points, ranging from 0.14 to 0.29 [29, 30]. In the current complementary study, we further explored T_V_ to derive various novel indicators of CR based on variability in T_V_, which, to our knowledge, has not been done in pigs or any other livestock species.

Animals that can better regulate their body temperature tend to have enhanced welfare and performance [31, 32] and are considered to be more heat-tolerant. In this context, CR is attributed to their ability to withstand environmental disturbances with minimal impact or to quickly return to their pre-disturbance state [16, 19, 20]. Most studies that evaluate resilience in pigs have focused on variability in feed efficiency, body weight, and disease challenges [20, 21, 33]. Therefore, the primary objectives of this study were to (1) derive novel CR indicators based on automatically recorded T_V_; (2) estimate the variance components and genetic parameters for the CR indicators, shoulder skin surface temperature (TSS), and RR; (3) estimate the genetic correlations between CR indicators and reproductive traits; and (4) evaluate HS-related physiological performance and BCS of animals with divergent genomic estimated breeding values (GEBV) for selected CR indicators.

## Methods

### Datasets

All live animal data collection procedures were approved by the Purdue University Animal Care and Use Committee (Protocol #1912001990). The variables and data collection procedures, genotype information, and quality control processes are described in great detail in Johnson et al. [28], Freitas et al. [29], and Wen et al. [30]. Briefly, the T_V_ of 1381 lactating sows (parities 2 to 7; Large White × Landrace) was measured automatically every 10 min from June 5th, 2021, to July 30th, 2021, using a vaginally implanted thermochron data recorder [28]. An average genomic relationship coefficient of 2.90 × 10^–05^ [standard deviation (SD) = 0.005] among the sows included in this study was calculated based on the $\mathbf{G}$ matrix. Ear (TES), shoulder (TSS), rump (TRS), and tail (TTS) surface temperatures, and RR were collected every day at 8:00 am, 12:00 pm, 4:00 pm, and 8:00 pm [28]. The total number of piglets born alive (LB), total number of piglets born (TB), and number of pigs weaned (PW) were also recorded by the farm employees [28]. All sows were genotyped using the PorcineSNP50K Bead Chip (Illumina, San Diego, CA, USA). Quality control of the genomic information was done as previously described by Wen et al. [30]. The environmental conditions (ambient temperature and humidity) within the barn were automatically recorded every five minutes [28].

### Defining climatic resilience indicators

Indicators of CR were developed by analyzing the pattern of the fluctuations in T_V_. In this study, a lower variability in T_V_ and an ability to quickly return to a normal state after changes due to heat stress indicates better CR. Table 1 presents the abbreviations and definitions of all the CR indicators derived from Tv data in this study. First, the deviations between an observed value and the average or median value from moving windows containing six continuous observations with a 10-min interval were calculated. The natural log-transformed variance of deviations (LnVar), lag-1 autocorrelation (${\text{Autocor}}=\frac{{\sum }_{{\text{t}}=1}^{{\text{n}}-1}({{\text{x}}}_{{\text{t}}}-\overline{{\text{x}} })({{\text{x}}}_{{\text{t}}+1}-\overline{{\text{x}} })}{{\sum }_{{\text{t}}=1}^{{\text{n}}}{({{\text{x}}}_{{\text{t}}}-\overline{{\text{x}} })}^{2}}$, where ${{\text{x}}}_{{\text{t}}}$ represents the deviation at time point ${\text{t}}$ and $n$ is the total number of time points), and skewness of deviations (Skew) were calculated based on the deviations described before, as previously suggested by Poppe et al. [18]. The HS threshold values for T_V_ were calculated using a breakpoint analysis and the model described by Johnson et al. [28], and found to be 39.75 °C for individuals in mechanically-ventilated barns and 39.78 °C for those in naturally-ventilated barns. The HS threshold value represents a critical temperature level at which lactating sows begin to experience HS. The other derived traits include the highest Tv (Max_Tv_) of each measurement day for each individual (i.e., repeated records per animal); and HS duration (HSD), which is the duration of the period during which the Tv of each individual remains above the HS threshold value for each measurement day. We also derived two traits corresponding to the normalized median ($\tt Nor\_medvar$) or average T_V_ ($\tt Nor\_avevar$) multiplied by the normalized T_V_ variance on the population level as follows:

$${{\text{Nor}}\_{\text{medvar}}}_{{\text{i}}}=\frac{{{\text{Med}}}_{{\text{i}}}-{{\text{Med}}}_{{\text{min}}}}{{{\text{Med}}}_{{\text{max}}}-{{\text{Med}}}_{{\text{min}}}}\times \frac{{{\text{Var}}({\text{Tv}})}_{{\text{i}}}-{{\text{Var}}({\text{Tv}})}_{{\text{min}}}}{{{\text{Var}}({\text{Tv}})}_{{\text{max}}}-{{\text{Var}}({\text{Tv}})}_{{\text{min}}}},$$

$${\text{and}}\, {{\text{Nor}}\_{\text{avevar}}}_{{\text{i}}}=\frac{{{\text{Ave}}}_{{\text{i}}}-{{\text{Ave}}}_{{\text{min}}}}{{{\text{Ave}}}_{{\text{max}}}-{{\text{Ave}}}_{{\text{min}}}}\times \frac{{{\text{Var}}({\text{Tv}})}_{{\text{i}}}-{{\text{Var}}({\text{Tv}})}_{{\text{min}}}}{{{\text{Var}}({\text{Tv}})}_{{\text{max}}}-{{\text{Var}}({\text{Tv}})}_{{\text{min}}}},$$

where ${{\text{Med}}}_{{\text{i}}}$, ${{\text{Ave}}}_{{\text{i}}}$, and ${{\text{Var}}({\text{Tv}})}_{{\text{i}}}$ represent the median, average, and variance of T_V_ for individual ${\text{i}}$, ${{\text{Ave}}}_{{\text{min}}}$ and ${{\text{Ave}}}_{{\text{max}}}$ are the minimum and maximum median T_V_, ${{\text{Med}}}_{{\text{min}}}$ and ${{\text{Med}}}_{{\text{max}}}$ are the minimum and maximum median T_V_, and ${{\text{Var}}({\text{Tv}})}_{{\text{min}}}$ and ${{\text{Var}}({\text{Tv}})}_{{\text{max}}}$ are the minimum and maximum T_V_ variance, respectively.

**Table 1:** Description of the climatic resilience indicators derived from automatically-recorded vaginal temperature in lactating sows

Indicator	Description	Interpretation
LnVar(Ave)	Log-transformed variance of the deviations between observed and the average values from moving windows^a^	Climateic resilient animals (minimally influenced by disturbances or with a fast recovery) exhibit lower LnVar(Ave). The variance of the deviations of time points from an average moving window reflects the severity of environmental perturbations, as well as the individual's rate of recovery
Autocor(Ave)	Lag-1 autocorrelation of the deviations between the average values from moving windows	Climatic resilient animals exhibit an autocorrelation close 0. It shows the association between the deviations between observed and the average value from moving windows
Skew(Ave)	Skewness of the deviations between the average values from moving windows	Climatic resilient animals exhibit a skewness to 0. It shows the asymmetry of the deviations between observed and the average value from moving windows. This indicator captures the severity and direction of environmental perturbations that an individual experiences
LnVar(Med)	Log-transformed variance of the deviations between the median values from moving windows	Similar to the LnVar(Ave)
Autocor(Med)	Lag-1 autocorrelation of the deviations between the median values from moving windows	Similar to the Autocor(Ave)
Skew(Med)	Skewness of the deviations between the median values from moving windows	Similar to the Skew(Ave)
HSU_A_	Sum of the Tv^b^ values above the heat stress (HS) threshold^c^ during the whole data collection period	Climatic resilient animals exhibited low(er) HSU_A_ values. In contrast, climatic sensitive animals show a high(er) value for HSU_A_. This measure captures the severity and duration of environmental perturbations
HSU_B_	Sum of the Tv values below the HS threshold during the whole data collection period	Measured in negative values. Climatic resilient animals tend to have a lower HSU_B_. Climatic sensitive animals exhibit higher values up to zero (superior limit for this trait). The HSU_B_ indicator effectively captures the severity and duration of environmental perturbations
Nor_medvar	Normalized median T_V_ multiplied by the normalized T_V_ variance	Climatic resilient animals tend to have a low(er) Nor_medvar value. In contrast, climatic sensitive animals show a high(er) value in Nor_medvar. This measure captures the severity of environmental perturbations
Nor_avevar	Normalized average T_V_ multiplied by the normalized T_V_ variance	Similar to Nor_medvar
HSD	Length of time during which the body temperature remains above the HS threshold value for each collection day	Climatic resilient animals tend to have a low(er) HSD value. In contrast, climatic sensitive animals show a high(er) value in HSD. This measure effectively captures the duration of environmental perturbations
Max_Tv_	The highest Tv of each measurement day	Climatic resilient animals tend to have a low(er) Max_Tv_ value. In contrast, climatic sensitive animals show a high(er) value in Max_Tv_. This measure captures the severity of environmental perturbations
Slope_In_	Slope for the increase of the Tv	Climatic resilient animals tend to have a low(er) Slope_In_ value. In contrast, climatic sensitive animals show a high(er) value in Slope_In_. This measure is a function of the severity and duration of environmental perturbations
Slope_De_	Slope for the decrease of the Tv	Positive value. Climatic resilient animals tend to have a high(er) Slope_De_ value. In contrast, climatic sensitive animals show a low(er) value in Slope_De_. This measure depends on the severity and duration of environmental perturbations
RA_slope_	Log transformed ratio of Slope_In_ to Slope_De_	Positive value. Climatic resilient animals tend to have a low(er) RA_slope_ value. In contrast, climatic sensitive animals show a high(er) value in RA_slope_. This measure effectively captures the severity and duration of environmental perturbations experienced by an individual

^a^The moving windows used in this study contained six continuous observations with a 10-min interval in between

^b^Automatically-recorded vaginal temperature

^c^Heat stress threshold: the heat stress threshold value for individuals under different ventilation treatments (mechanical ventilation: 39.75 °C and natural ventilation: 39.78 °C) was estimated as previously described in Johnson et al. [23]

**Fig. 1:**
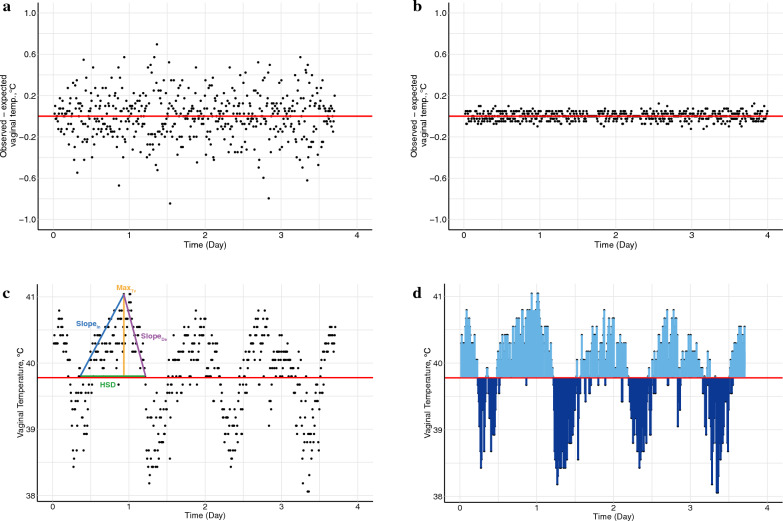
Example of the derivation of climate resilience indicators. Deviations in observed versus expected vaginal temperature in two example individuals, pig A (panel **a**), pig B (panel **b**). Panel **c** shows how the measures HSD, Slope_In_, Slope_De_, and Max_Tv_ were obtained for pig A. In panel** d**, the light blue area represents the HSU_A_ for pig A, which is the sum of the vaginal temperatures above the heat stress threshold, while the dark blue area represents the HSU_B_ for pig A, which is the sum of the vaginal temperatures below the heat stress threshold. HSD: length of time during which the body temperature of each individual remains above the heat stress threshold value for each collection day; Slope_In_: slope of the increase in Tv; Slope_De_: slope of the decrease in Tv. Max_Tv_: the highest T_V_ of each collection day for each individual; HSU_A_: sum of the Tv values above the HS threshold during the whole data collection period; HSU_B_: sum of the Tv values below the HS threshold during the whole data collection period

Two additional traits were derived based on the total deviations between T_V_ and HS threshold values, which were calculated by summing up the T_V_ values above (${{\text{HSU}}}_{{\text{A}}}$) or below (${{\text{HSU}}}_{{\text{B}}}$) the HS threshold throughout the entire data collection period as follows:

$${{\text{HSU}}}_{{\text{A}}}=\sum_{T_{V_t} >\text{HS threshold}}\left({{\text{Tv}}}_{{\text{t}}}-\mathrm{HS\,threshold}\right),$$

$${\text{HSU}}_{{\text{B}}}=\sum_{T_{V_t}<\text{HS threshold}}\left({{\text{Tv}}}_{{\text{t}}}-\mathrm{HS\,threshold}\right),$$

where ${{\text{Tv}}}_{{\text{t}}}$ is the T_V_ at time point ${\text{t}}$. Furthermore, the slope for the increase (Slope_In_) or decrease (Slope_De_) of the T_V_ was calculated as (see Fig. 1c):

$${{\text{Slope}}}_{{\text{In}}}=\left\{\begin{array}{l}\frac{{{{\text{T}}}_{{\text{V}}}}_{{\text{Max}}}-{{{\text{T}}}_{{\text{V}}}}_{{\text{Min}}}}{\mathrm{time\,at }{{{\text{T}}}_{{\text{V}}}}_{{\text{Max}}}-\mathrm{ time\,at }{{{\text{T}}}_{{\text{V}}}}_{{\text{Min}}}}, \qquad \quad if\, {{{\text{T}}}_{{\text{V}}}}_{{\text{Min}}}>HS\,threshold\\ \frac{{{{\text{T}}}_{{\text{V}}}}_{{\text{Max}}}-\mathrm{HS\,threshold}}{\mathrm{time\,at }{{{\text{T}}}_{{\text{V}}}}_{{\text{Max}}}-\mathrm{ time\,at }{{{\text{T}}}_{{\text{V}}}}_{\mathrm{HS\,threshold}}}, \quad if\, {{{\text{T}}}_{{\text{V}}}}_{{\text{Min}}}\le HS\,threshold\end{array},\right.$$

$${\text{and}}\, {{\text{Slope}}}_{{\text{De}}}=\left\{\begin{array}{l}\left|\frac{{{{\text{T}}}_{{\text{V}}}}_{{\text{Min}}}-{{{\text{T}}}_{{\text{V}}}}_{{\text{Max}}}}{\mathrm{time\,at }{{{\text{T}}}_{{\text{V}}}}_{{\text{Max}}}-\mathrm{ time\,at }{{{\text{T}}}_{{\text{V}}}}_{{\text{Min}}}}\right|, \quad if \, {{{\text{T}}}_{{\text{V}}}}_{{\text{Min}}}>HS\,threshold\\ \left|\frac{{{{\text{T}}}_{{\text{V}}}}_{{\text{Max}}}-\mathrm{HS\,threshold}}{\mathrm{time\,at }{{{\text{T}}}_{{\text{V}}}}_{{\text{Max}}}-\mathrm{ time\,at }{{{\text{T}}}_{{\text{V}}}}_{{\text{Min}}}}\right|, \quad if \,{{{\text{T}}}_{{\text{V}}}}_{{\text{Min}}}\le HS\,threshold\end{array}.\right.$$

Most of the Slope_In_ and Slope_De_ values consistently occurred in pairs for each animal due to the circadian rhythms of the animals’ body temperature. The slope ratio was calculated as ${{\text{RA}}}_{{\text{slope}}}=\log\frac{{{\text{Slope}}}_{{\text{In}}}}{{{\text{Slope}}}_{{\text{De}}}}$ for each corresponding pair of these variables. Any Slope_In_ or Slope_De_ that did not have a corresponding pair was removed. For each CR indicator, potential outliers were discarded if they deviated by more than four SD from the mean. In summary, the derived CR indicators were grouped as: (1) deviation-based indicators (LnVar, Autocor, and Skew); (2) slope-based indicators (Slope_In_, Slope_De_, and RA_slope_), and (3) the other indicators (HSU_A_, HSU_B_, Nor_medvar, Nor_avevar, HSD, and Max_Tv_). The description of these indicators is provided in Fig. 1 and Table 1.

### (Co)variance components and estimation of genetic parameters

(Co)variance components and genetic parameters were calculated based on the Average Information Restricted Maximum Likelihood (AIREML) algorithm implemented in the BLUPf90 + family of programs [34, 35]. The fixed and random effects, and the number of records for each trait are in Table 2. Location, parity, and week of data collection were considered as fixed effects for Nor_medvar, Nor_avevar, and all deviation-based indicators. For the other indicators, only parity and week of data collection were considered as fixed effects. Single record animal models and repeatability animal models were fitted for the CR indicators with a single record and multiple records, respectively.

**Table 2:** Summary statistics and genetic models for climatic resilience indicators derived from automatically measured vaginal temperatures in lactating sows

Indicator^a^	N	Mean	SD	Median	Min	Max	Effects included in the mixed models	
							Fixed effects^b^	Random effects^c^
LnVar(Ave)	1366	0.007	0.005	0.005	0.002	0.029	Loc, Par, Week	a
Autocor(Ave)	1380	− 0.008	0.065	− 0.009	− 0.212	0.232	Loc, Par, Week	a
Skew(Ave)	1374	− 0.021	0.355	0.016	− 1.526	1.476	Loc, Par, Week	a
LnVar(Med)	1366	0.006	0.004	0.005	0.001	0.027	Loc, Par, Week	a
Autocor(Med)	1380	0.0640	0.070	0.064	− 0.157	0.279	Loc, Par, Week	a
Skew(Med)	1375	− 0.159	0.710	− 0.080	− 3.158	2.567	Loc, Par, Week	a
HSU_A_/ °C	1378	195.161	178.371	146.383	0	919.045	Par, Week	a
HSU_B_/ °C	1376	− 216.16	177.65	− 177.99	− 929.68	0	Par, Week	a
Nor_medvar	1374	0.132	0.075	0.120	0	0.453	Loc, Par, Week	a
Nor_avevar	1374	0.249	0.117	0.232	0	0.745	Loc, Par, Week	a
HSD/10 min	6034	74.387	41.350	75.000	0	144	Par, Week	a, pe
Max_Tv_/°C	6539	40.621	0.594	40.646	38.245	42.842	Par, Week	a, pe
Slope_In_	5077	1.503	0.419	1.367	0.7226	3.003	Par, Week	a, pe
Slope_De_	5837	3.467	0.026	3.473	3.363	3.477	Par, Week	a, pe
RA_slope_	4980	2.917	0.726	2.829	0.8447	4.851	Par, Week	a, pe

*N* number of records, *SD* standard deviation

^a^Trait abbreviations are described in Table 1

^b^Fixed effects: Loc, location of individual, which includes the barn and the room within the barn of the animal; Par, parity; Week, week of collection

^c^Random effects: a, additive genetic effect; pe, permanent environmental effect

The genomic relationship matrix ($\mathbf{G}$) was calculated as $\mathbf{Z}{\mathbf{Z}}^{\mathbf{^{\prime}}}/\sum 2pq$, where $p$ and $q$ are the frequency of the first and second alleles at each locus and $\mathbf{Z}$ is a matrix of genotypes centered for the allele frequencies. The heritability estimates for traits with single records, i.e. LnVar(Ave), Autocor(Ave), Skew(Ave), LnVar(Med), Autocor(Med), Skew(Med), HSU_A_, HSU_B_, Nor_medvar, and Nor_avevar, and both the heritability and repeatability estimates for traits with repeated records, i.e. HSD, Max_Tv_, Slope_In_, Slope_De_, RA_slope_ were calculated, respectively, as:

$${{\text{h}}}^{2}=\frac{{\widehat{\upsigma }}_{{\text{a}}}^{2}}{{\widehat{\upsigma }}_{{\text{a}}}^{2}+{\widehat{\upsigma }}_{{\text{e}}}^{2}},$$

$${{\text{h}}}^{2}=\frac{{\widehat{\upsigma }}_{{\text{a}}}^{2}}{{\widehat{\upsigma }}_{{\text{a}}}^{2}+{\widehat{\upsigma }}_{{\text{pe}}}^{2}+{\widehat{\upsigma }}_{{\text{e}}}^{2}},$$

$${\text{and}}\, {\text{r}}=\frac{{\widehat{\upsigma }}_{{\text{a}}}^{2}+{\widehat{\upsigma }}_{{\text{pe}}}^{2}}{{\widehat{\upsigma }}_{{\text{a}}}^{2}+{\widehat{\upsigma }}_{{\text{pe}}}^{2}+{\widehat{\upsigma }}_{{\text{e}}}^{2}},$$

where ${{\text{h}}}^{2}$, ${\text{r}}$, ${\widehat{\upsigma }}_{{\text{a}}}^{2}$, ${\widehat{\upsigma }}_{{\text{pe}}}^{2}$, ${\widehat{\upsigma }}_{{\text{e}}}^{2}$ are the estimates of the heritability, repeatability, additive genetic variance, permanent environmental variance, and residual variance, respectively. Phenotypic and genetic correlations among all the CR indicators, TSS, and RR were calculated using bivariate models with the BLUPf90 + family of programs [34, 35].

The GEBV of five selected CR indicators, i.e. Nor_medvar, Max_Tv_, HSD, HSU_A_, and HSU_B_, were calculated for each sow. The selection of these five CR indicators was based on their high genetic correlation with each other, which was positive and higher than 0.5. The GEBV accuracy was calculated as:$\sqrt{1- \frac{{SEP}^{2}}{{\widehat{\sigma }}_{a}^{2}}}$, where $SEP$ is the standard error of the prediction and ${\widehat{\sigma }}_{a}^{2}$ is the estimated additive genetic variance. Inbreeding was not considered in the calculation of GEBV accuracy since all the animals are F1 crosses. Furthermore, for the five selected CR indicators, we calculated the genetic correlations among the five indicators and three reproductive performance traits (LB, TB, and PW) and the Spearman rank correlations between their GEBV. Since HSU_A_ and HSU_B_ had the same ranking list (see Additional file 1 Table S1), only four of the five indicators (Nor_medvar, Max_Tv_, HSD, and HSU_B_) were kept for further analyses. The top 100 (6.10%) and bottom 100 (6.10%) individuals were selected based on their GEBV for these four CR indicators. Venn diagrams of the top and bottom 100 individuals for each indicator were created using the R package VennDiagram [36]. The HS-related physiological performance, including skin surface temperatures (TES, TSS, TRS, and TTS), RR, hair density (HD), and caliper body condition score (BCS_cal_), were compared for the top and bottom individuals. Only individuals with a GEBV accuracy greater than 0.60 for each indicator were included in this analysis. Statistically significant differences (*P* < 0.05) in HS-related physiological performance between the top and bottom individuals were determined using the Student t-test as implemented in the R software [37].

## Results and discussion

Table 2 shows the descriptive statistics for the 15 CR indicators derived in this study. For five of these (HSD, Max_Tv_, Slope_In_, Slope_De_, and RA_slope_), there were more than 4900 records because individuals had repeated records for each collection day. The other indicators with a single record were estimated using all the data throughout the entire collection period. The average Max_Tv_ was 40.62 °C, which significantly surpassed the HS temperature threshold (39.7 °C, [28]). In total, 49.09% of the T_V_ records exceeded the HS threshold in our dataset, which confirms that the sows were environmentally challenged during the data collection period. Furthermore, the mean HSD was 74.387/10 min-intervals, which means that the sows experienced an average HSD of 743.87 min (12.40 h), each day. These results indicate that the studied population was under chronic HS, which is further supported by the high average T_V_ (39.739 ± 0.758). Moreover, Slope_De_ had larger mean and median values than Slope_In_, which indicates that the T_V_ returned to the HS threshold levels generally more rapidly than the initial T_V_ increase triggered by the environmental stress.

### Heritability and repeatability estimates for climatic resilience indicators

To the best of our knowledge, this study is the first one that reports genetic parameters for CR indicators that are derived based on automatically recorded T_V_. Table 3 shows the heritability estimates (standard error, SE), repeatability estimates (SE), and mean GEBV accuracies (SD) for the 15 CR indicators. Their heritability estimates ranged from 0.000 ± 0.000 (RA_slope_) to 0.291 ± 0.047 (HSU_B_). The heritability estimates of the deviation-based indicators (LnVar, Autocor, and Skew) were low to moderate and these three CR indicators had moderate GEBV accuracies (Table 3). Regardless of whether mean or median values were used to define the CR indicators for the methods based on deviations, LnVar had consistently higher heritability estimates than Autocor. This observation aligns with previous resilience studies using daily milk yield [17, 18] and step count [16] in dairy cattle. In most studies, LnVar has low heritability estimates (from 0.01 to 0.14), regardless of whether single or repeated records per individual were used, e.g. in pigs [21, 38], dairy cattle [17, 39], or aquatic species [20]. Skew had the lowest heritability among the deviation-based indicators in our study, which aligns with the resilience studies using longitudinal egg production in Bedere et al. [22] and milk yield data in Poppe et al. [18]. Although Skew can indicate the direction (positive or negative) and severity of disturbances [40], it might be more sensitive to outliers and less practical for breeding purposes. However, these deviation-based resilience indicators do not always show the same trend. When the variability in body weight was analyzed in chickens, the heritability estimates of LnVar, Autocor, and Skew indicators were all close to 0.100 [30, 41], which indicates that body weight is a moderate-term response to environmental disturbances [21] and cannot respond as quickly as milk yield or body temperature to environmental disturbances and, therefore, may not quantify short-term resilience with a high accuracy [21].

**Table 3:** Heritability (SE), repeatability (SE), and mean GEBV accuracy (SE) for novel climate resilience indicators derived from automatically measured vaginal temperatures in lactating sows

Indicator^a^	Heritability (SE)	Repeatability (SE)	Mean GEBV accuracy (SE)
LnVar(Ave)	0.185 (0.0003)	–	0.555 (0.057)
Autocor(Ave)	0.154 (0.042)	–	0.513 (0.059)
Skew(Ave)	0.146 (0.041)	–	0.504 (0.055)
LnVar(Med)	0.196 (0.041)	–	0.616 (0.042)
Autocor(Med)	0.109 (0.039)	–	0.453 (0.056)
Skew(Med)	0.084 (0.037)	–	0.409 (0.059)
HSU_A_	0.258 (0.046)	–	0.610 (0.065)
HSU_B_	0.291 (0.047)	–	0.634 (0.059)
Nor_medvar	0.230 (0.047)	–	0.588 (0.057)
Nor_avevar	0.205 (0.046)	–	0.566 (0.061)
HSD	0.201 (0.033)	0.547 (0.142)	0.644 (0.061)
Max_Tv_	0.203 (0.032)	0.538 (0.097)	0.652 (0.066)
Slope_In_	0.0004 (< 0.001)	0.0032 (0.006)	0.337 (0.065)
Slope_De_	0.008 (0.011)	0.054 (0.019)	0.111 (0.061)
RA_slope_	0.552 × 10^–4^ (0.121 × 10^–5^)	0.125 × 10^–4^ (0.621 × 10^–5^)	0.123 (0.056)

^a^Trait abbreviations are described in Table 1

The slope-based indicators, including Slope_In_, Slope_De_, and RA_slope_, had low heritability estimates (from 0 to 0.008 ± 0.011), low repeatability estimates (from 0 to 0.054 ± 0.019), and low to moderate mean GEBV accuracies (from 0.111 ± 0.061 to 0.337 ± 0.065). The other six CR indicators (HSD, Max_Tv_, Nor_medvar, Nor_avevar, HSU_A_, and HSU_B_) had moderate heritability estimates (from 0.201 ± 0.033 to 0.291 ± 0.047) and GEBV accuracies (from 0.566 ± 0.061 to 0.652 ± 0.066). The heritability estimates of these six CR indicators align with the results reported for T_V_ heritability in our previous study [29]. In addition, both HSD (0.547 ± 0.142) and Max_Tv_ (0.538 ± 0.097) exhibited moderate repeatability estimates, which suggests that animals with longer HSD and higher Max_Tv_ values are more likely to show higher Tv values over multiple measurements. Since Nor_medvar, HSU_A_, HSU_B_, HSD, and Max_Tv_ showed relatively higher heritabilities than the other indicators and do not rely on complex calculations, these traits might be more useful in pig breeding programs.

Different breeds or even lines might exhibit varying levels of CR due to their distinct genetic characteristics or to the effects of long-term artificial selection. Cuellar et al. [42] reported lower average T_V_ and significantly lower Max_Tv_ in crossbred animals (mostly Brown Swiss × Holstein F1 animals) compared to Holstein and Brown Swiss individuals . Similar results were found in sheep [43] and pigs [44]. These findings suggest that the effects of breed or line on CR should be further investigated in future studies.

### Phenotypic and genetic relationships

To investigate the relationship among CR indicators, we used bivariate models to calculate their phenotypic and genetic correlations, which are presented in Tables 4 and 5, respectively. Table 5 also shows the genetic correlation of CR indicators with HS-related physiological responses (TSS and RR). The phenotypic correlations between each of the CR indicators ranged from − 0.34 ± 0.08 [between LnVar(Med) and Autocor(Med)] to 0.99 ± 0.13 [between LnVar(Med) and LnVar(Ave)]. In contrast, the genetic correlations ranged from − 0.72 ± 0.11 [between Skew(Med) and Max_Tv_] to 0.99 ± 0.13 [between Skew(Med) and Skew(Ave)].

**Table 4:** Phenotypic correlations (above the diagonal) and standard error (below the diagonal) among the climate resilience indicators derived from automatically measured vaginal temperatures in lactating sows

Indicator^a^	LnVar(Ave)	Autocor (Ave)	Skew (Ave)	LnVar (Med)	Autocor (Med)	Skew (Med)	HSU_A_	HSU_B_	Nor_medvar	Nor_avevar	HSD	Max_Tv_	Slope_In_	Slope_De_	RA_slope_
LnVar(Ave)		− 0.200	− 0.144	0.990	− 0.319	− 0.123	− 0.200	− 0.174	0.125	0.224	− 0.233	0.017	0.155	0.207	0.040
Autocor(Ave)	0.102		0.181	− 0.256	0.765	0.144	− 0.086	0.133	− 0.111	− 0.183	0.005	− 0.024	0.021	− 0.004	0.035
Skew(Ave)	0.075	0.016		− 0.149	0.117	0.914	− 0.193	− 0.162	− 0.242	− 0.177	− 0.086	− 0.115	0.014	− 0.026	− 0.015
LnVar(Med)	0.137	0.021	0.041		− 0.347	− 0.123	− 0.082	− 0.179	0.116	0.214	0.206	0.010	0.149	0.206	0.040
Autocor(Med)	0.122	0.031	0.027	0.085		0.057	0.017	0.135	− 0.136	− 0.212	0.052	− 0.035	− 0.024	− 0.003	0.024
Skew(Med)	0.089	0.028	0.106	0.032	0.006		− 0.190	− 0.171	− 0.220	− 0.144	− 0.069	− 0.107	0.008	− 0.005	− 0.012
HSU_A_	0.093	0.091	0.019	0.018	0.015	0.041		0.667	0.548	0.204	0.598	0.628	− 0.064	− 0.039	− 0.096
HSU_B_	0.097	0.028	0.015	0.026	0.026	0.037	0.093		0.385	0.013	0.518	0.536	− 0.195	− 0.024	− 0.115
Nor_medvar	0.074	0.041	0.009	0.037	0.028	0.055	0.074	0.137		0.868	0.374	0.497	0.016	0.011	− 0.045
Nor_avevar	0.083	0.039	0.011	0.021	0.042	0.033	0.037	0.008	0.122		0.136	0.323	0.046	0.036	− 0.014
HSD	0.109	0.007	0.007	0.023	0.013	0.014	0.079	0.091	0.056	0.021		0.770	− 0.338	− 0.228	− 0.128
Max_Tv_	0.005	0.012	0.063	0.009	0.011	0.039	0.082	0.103	0.071	0.042	0.083		− 0.071	− 0.065	− 0.158
Slope_In_	0.074	0.009	0.004	0.011	0.008	0.005	0.026	0.052	0.007	0.014	0.041	0.016		− 0.025	0.709
Slope_De_	0.116	0.003	0.010	0.015	0.002	0.002	0.013	0.011	0.004	0.008	0.037	0.014	0.011		− 0.221
RA_slope_	0.018	0.011	0.007	0.008	0.011	0.007	0.046	0.043	0.013	0.005	0.022	0.031	0.049	0.039	

^a^Trait abbreviations are described in Table 1

**Table 5:** Genetic correlations (above the diagonal) and standard error (below the diagonal) among the climate resilience indicators derived from automatically measured vaginal temperatures and shoulder skin surface temperature and respiration rate in lactating sows

Indicator^a^	LnVar (Ave)	Autocor (Ave)	Skew (Ave)	LnVar (Med)	Autocor (Med)	Skew (Med)	HSU_A_	HSU_B_	Nor_medvar	Nor_avevar	HSD	Max_Tv_	Slope_In_	Slope_De_	RA_slope_	TSS	RR
LnVar(Ave)		− 0.223	− 0.609	0.998	− 0.368	− 0.678	− 0.107	− 0.164	0.184	0.397	− 0.277	0.018	0.580	0.360	0.677	− 0.068	− 0.068
Autocor(Ave)	0.052		0.355	− 0.270	0.939	0.275	0.114	0.375	− 0.095	− 0.289	0.112	0.056	0.218	0.415	− 0.585	− 0.349	− 0.188
Skew(Ave)	0.085	0.105		− 0.621	0.354	1.000	− 0.501	− 0.451	− 0.514	− 0.464	− 0.415	− 0.603	− 0.449	− 0.572	− 0.473	− 0.187	0.045
LnVar(Med)	0.124	0.089	0.134		− 0.392	− 0.704	− 0.097	− 0.148	0.155	0.372	− 0.218	− 0.053	0.848	0.372	0.713	− 0.069	− 0.088
Autocor(Med)	0.075	0.186	0.098	0.048		0.220	0.259	0.511	0.072	− 0.209	0.178	0.111	− 0.364	0.433	− 0.665	− 0.142	− 0.062
Skew(Med)	0.097	0.043	0.132	0.094	0.018		− 0.620	− 0.559	− 0.635	− 0.597	− 0.485	− 0.718	− 0.493	− 0.584	− 0.569	− 0.127	0.095
HSU_A_	0.049	0.056	0.069	0.063	0.024	0.098		0.890	0.781	0.395	0.990	0.875	− 0.090	− 0.104	− 0.413	0.377	0.213
HSU_B_	0.078	0.058	0.085	0.015	0.118	0.067	0.154		0.508	0.035	0.981	0.800	0.008	− 0.069	− 0.373	0.139	0.079
Nor_medvar	0.102	0.089	0.074	0.049	0.076	0.108	0.096	0.056		0.886	0.883	0.944	− 0.363	− 0.084	− 0.628	0.432	0.502
Nor_avevar	0.098	0.068	0.052	0.061	0.082	0.094	0.064	0.011	0.106		0.480	0.698	− 0.118	− 0.148	− 0.585	0.381	− 0.069
HSD	0.057	0.097	0.046	0.047	0.071	0.069	0.201	0.098	0.119	0.062		0.907	− 0.405	− 0.315	− 0.151	0.462	0.290
Max_Tv_	0.022	0.031	0.103	0.009	0.063	0.111	0.099	0.079	0.203	0.093	0.094		− 0.010	− 0.024	− 0.162	0.478	0.331
Slope_In_	0.065	0.028	0.068	0.097	0.058	0.071	0.012	0.009	0.094	0.029	0.067	0.013		0.220	− 0.221	− 0.478	− 0.143
Slope_De_	0.048	0.084	0.081	0.064	0.123	0.083	0.021	0.013	0.026	0.034	0.081	0.008	0.031		− 0.079	− 0.002	0.280
RA_slope_	0.098	0.114	0.122	0.114	0.101	0.079	0.061	0.032	0.018	0.019	0.013	0.019	0.024	0.008		− 0.352	0.775
TSS	0.013	0.029	0.094	0.009	0.015	0.026	0.024	0.008	0.079	0.046	0.069	0.048	0.054	0.006	0.061		0.240
RR	0.025	0.041	0.081	0.007	0.019	0.057	0.057	0.011	0.098	0.015	0.029	0.039	0.040	0.011	0.094	0.016	

^a^The trait abbreviations are described in Table 1

Five pairs of CR indicators, i.e. LnVar(Ave)–LnVar(Med), Autocor(Ave)–Autocor(Med), Skew(Ave)–Skew(Med), HSU_A_–HSU_B_, and Nor_medvar–Nor_avevar, exhibited high positive phenotypic ($\ge$ 0.667) and genetic ($\ge$ 0.886) correlations. LnVar was moderately to highly negatively genetically correlated with both Skew and Autocor (Table 5), which is further supported by the positive and moderate correlation observed between Skew and Autocor. The deviation-based indicators were genetically correlated with other CR indicators with low to high estimates, e.g. for LnVar (ranging from − 0.277 ± 0.057 to 0.397 ± 0.098), Autocor (from − 0.289 ± 0.068 to 0.511 ± 0.118), and Skew (from − 0.718 ± 0.111 to − 0.062 ± 0.046). These correlations were consistent regardless of whether average or median values were used to calculate the CR indicator value. This is in line with the results from Poppe et al. [18]. Moreover, the data that are used have a greater impact on the genetic correlation values than the method employed to create these deviation-based CR indicators [16]. For instance, with milk yield data, LnVar was negatively correlated with Autocor [18], but with step count data in dairy cattle [16] and body weight data in pigs [21], LnVar was positively genetically correlated with Autocor. The genetic correlations between LnVar and other resilience indicators, such as weighted occurrence frequency of yield perturbations derived from milk yield data in Holstein cattle, were low and ranged from − 0.274 ± 0.098 to − 0.088 ± 0.176 [17], while those between LnVar and indicators derived from step counts in Holstein cattle were high and ranged from − 0.93 ± 0.022 to 0.94 ± 0.032 [16].

The genetic correlations of the CR indicators with TSS ranged from − 0.349 ± 0.029 to 0.478 ± 0.048, while those with RR ranged from − 0.188 ± 0.041 to 0.502 ± 0.098. Interestingly, the deviation-based indicators were negatively or not correlated with TSS (− 0.068 ± 0.013 to − 0.349 ± 0.029) and RR (− 0.188 ± 0.041 to 0.095 ± 0.057). Thus, selecting for lower values of deviation-based CR indicators might not affect TSS and RR response. In contrast, the non-deviation-based indicators mainly exhibited positive genetic correlations with TSS (from 0.139 ± 0.008 to 0.478 ± 0.048) and RR (from − 0.069 ± 0.015 to 0.502 ± 0.098), which is consistent with our previous results on the genetic correlations between Tv and TSS (0.25 to 0.76) and RR (0.16 to 0.42) [29].

Moderate genetic correlation values were observed among HSD, Max_Tv_, Nor_medvar, HSU_A_, and HSU_B_ (from 0.508 ± 0.056 to 0.990 ± 0.201). Four of them, i.e. HSD, Max_Tv_, HSU_A_, Nor_medvar were moderately genetically correlated with both TSS (from 0.377 ± 0.024 to 0.478 ± 0.048) and RR (from 0.213 ± 0.057 to 0.502 ± 0.098). Notably, the absolute genetic correlation values of TSS with all the CR indicators were, for the most part, higher than those of RR with all the CR indicators, except for Nor_medvar.

As far as we know, this is the first study to investigate the phenotypic and genetic relationship between CR and reproductive performance in lactating sows. The genetic correlations between CR indicators (Nor_medvar, HSU_B_, HSD, and Max_Tv_) and three reproductive performance traits (LB, TB, and PW) ranged from − 0.733 to − 0.175, − 0.261 to 0.086, and − 0.434 to − 0.169, respectively (Table 6), which indicate that genetically improving CR in pigs is expected to result in better reproductive performance. The genetic correlations of LB with TB, LB with PW, and TB with PW0.169 were equal to 0.970, 0.120, and 0.169, respectively.

**Table 6:** Genetic correlations (standard error) between four of the climatic resilience indicators (HSU_B_, Nor_medvar, HSD, and Max_Tv_) and three reproductive performance traits in lactating sows

Indicators^a^	LB	TB	PW
HSU_B_	− 0.175 (0.041)	0.086 (0.009)	− 0.169 (0.019)
Nor_medvar	− 0.535 (0.066)	− 0.170 (0.045)	− 0.434 (0.052)
HSD	− 0.175 (0.037)	− 0.089 (0.013)	− 0.213 (0.034)
Max_Tv_	− 0.245 (0.034)	− 0.261 (0.042)	− 0.288 (0.038)

^a^Trait abbreviations are described in Table 1

*LB* total number of piglets born alive, *TB* total number of piglets born, *PW* number of pigs weaned

### Ranking of sows based on the GEBV of CR indicators

**Fig. 2:**
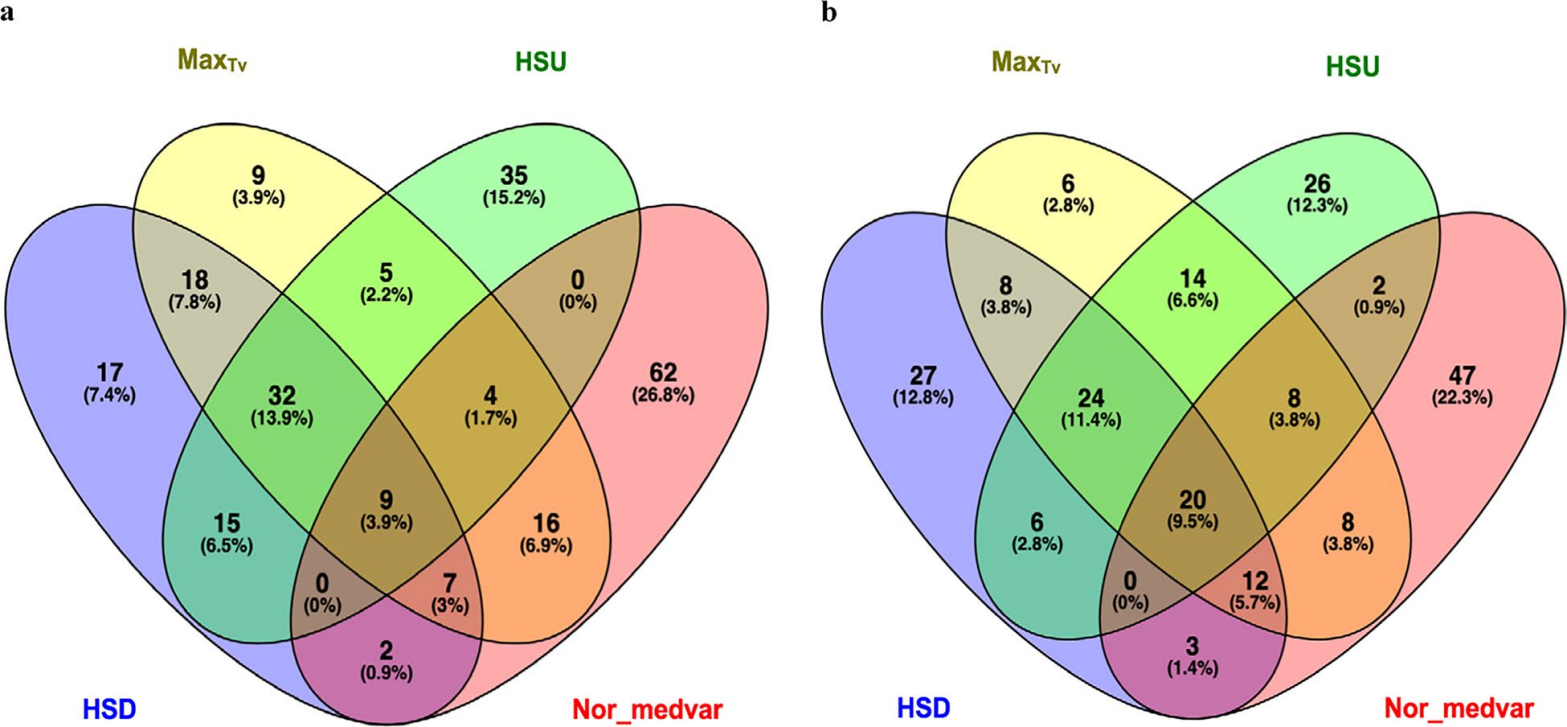
Venn diagram of the count and overlapping of (**a**) the top 100 individuals and (**b**) the bottom 100 individuals identified based on four of the climatic resilience indicators, i.e. HSD, Max_Tv_, HSU_B_, Nor_medvar. HSD: length of time during which the body temperature of each individual remains above the heat stress threshold value for each collection day; Max_Tv_: the highest T_V_ of each collection day for each individual; Nor_medvar: normalized median T_V_ multiplied by the normalized T_V_ variance; HSU_B_: the sum of the Tv values below the HS threshold during the whole data collection period

The overlap among the top (most heat-sensitive) and bottom (most heat-resilient) 100 individuals that were selected based on their GEBV for various CR indicators is shown in Fig. 2. The same animals were identified for HSU_A_ and HSU_B_, thus, only HSU_B_ is shown in Fig. 2. Nine individuals ranked in the top 100 across all four indicators, 43 ranked in the top 100 for three CR indicators, 56 ranked in the top 100 for any two indicators, and 123 for any one indicator.

Regarding the bottom 100 individuals, 20 individuals ranked for all four indicators, 44 individuals for three indicators, 31 individuals for at least two indicators, and 106 individuals for at least one CR indicator. Notably, high similarities were observed among the ranking results based on the GEBV for different CR indicators, with 77.5% and 68.2% of the individuals listed as the top 100 and bottom 100 for at least three CR indicators, respectively. This finding is supported by the moderate to high genetic correlations (from 0.508 ± 0.056 to 0.990 ± 0.201) and moderate to high Spearman rank correlations between the GEBV (0.431–0.908) for the five CR indicators.

### Physiological performance of climate-sensitive and -resilient animals

Table 7 presents the mean skin surface temperatures (TES, TSS, TRS, and TTS), PS, BCScal, HD, and RR for all the top and bottom individuals selected based on their GEBV for each of the four following CR indicators: Max_Tv_, HSU_B_, HSD, and Nor_medvar. The group of top (heat-sensitive) individuals had significantly higher mean skin surface temperatures (TES, TSS, TRS, and TTS), RR, PS, and HD compared to the group of bottom (heat-resilient) individuals (*P* < 0.05). Previous research showed that in sheep, goats [45–48], and beef cattle [49], coping mechanisms such as lowering the body temperature and RR have evolved to counteract the negative effects of hot environmental conditions and better adapt to harsh environments. In addition, BCS_cal_ values for the group of top individuals were significantly lower than those for the group of bottom individuals (*P* < 0.05). These findings indicate that animals with lower values for HS-related responses (skin surface temperatures, RR, PS, and HD) and a higher body condition score could be accurately selected based on the CR indicators under HS conditions. Previous research highlighted how HS might significantly impact body composition-related traits, such as off-test weight, muscle depth, and backfat thickness [14]. These traits can affect the body shape of animals and how they gain and lose heat. Animals with long, thin bodies tend to experience reduced heat gain and greater heat loss [50]. Lactating sows with higher CR indicators can exhibit relatively better HS-related response and have higher BCS. Our results also show a favorable genetic association between some CR indicators and reproductive performance. However, the relationship between these CR indicators and other economically important traits should be explored further to identify the optimal CR indicators. Balanced breeding and selection index should still be considered in pig breeding programs for improving the sustainability of pig production systems.

**Table 7:** Mean heat stress related performance (SD) between all the top and bottom individuals selected based on the climate resilience indicators derived from automatically measured vaginal temperatures in lactating sows

Group^a^	TES	TSS	TRS	TTS	PS	BCS_cal_	HD	RR
Top_individuals (heat sensitive)	36.867 (1.078)	36.612 (1.077)	37.360 (0.915)	37.028 (0.941)	1.025 (0.603)	11.554 (2.119)	1.078 (0.631)	73.976 (29.029)
Bottom_individuals (heat resilient)	36.597 (1.061)	36.257 (1.101)	37.027 (0.930)	36.738 (0.956)	0.874 (0.590)	12.224 (1.789)	1.008 (0.650)	68.094 (26.364)

*TES* ear skin temperature (°C), *TSS* shoulder skin temperature (°C), *TRS* rump skin temperature (°C), *TTS* tail skin temperature (°C), *PS* panting score, *BCS*_*cal*_ caliper body condition score, *HD* hair density, *RR* respiration rate

^a^Group: Top_individuals means all the selected 100 individuals with the highest mean GEBV and Bottom_ individuals means all the 100 individuals with the lowest mean GEBV of resilience indicators (Max_Tv_, HSU, HSD, and Nor_medvar)

### Challenges and implications

Several important points need to be considered in future studies. First, additional longitudinal T_V_ records from lactating sows should be collected under thermoneutral conditions (e.g., during the spring or winter seasons), on individuals raised in a wider range of environmental conditions, and from different lines and breeds. The use of more diverse phenotypic datasets will contribute to the validation of the CR indicators proposed here. Second, there is a need for more complete investigations into the genetic relationships between CR indicators and other economically important traits, such as body weight, carcass composition, feed intake, feed efficiency, longevity, and other health and welfare traits. This would enable a comprehensive evaluation of the additional value of the CR indicators proposed here. In addition, since feed intake and feeding patterns can be affected by HS, thereby causing reduction in most productive performance traits [51], it would be relevant to investigate their relationship with CR indicators and the impact of fitting them as variables in the CR models. Furthermore, as our analyses included genetic information from F1 crossbreed sows (Large White and Landrace) raised under commercial conditions, it would be interesting to re-evaluate the usefulness of the proposed CR indicators based on data collected in nucleus herds and other independent populations. It is also necessary to further evaluate the breeding goals related to CR based on T_V_ since selecting for reduced body temperature variability may have additional physiological implications, and to evaluate CR during lactation and other pig life stages.

In this study, we have demonstrated that the developed CR indicators can be applied to various longitudinal traits such as feed intake, milk yield, and body weight but we also highlight the multifaceted advantages of enhancing livestock resilience, among which increased productivity, improved reproductive performance, and enhanced animal welfare. Our results contribute to the understanding of swine CR and offer valuable guidance to breeders and geneticists for refining breeding programs and augmenting overall resilience in animals.

## Conclusions

To our knowledge, this is the first study that defines and investigates the genetic background of climatic resilient traits using automatically-recorded vaginal temperature data from lactating sows. Most of the CR indicators defined in this study are heritable and could be used to select pigs for enhanced CR, especially during lactation. High genetic correlations and Spearman rank correlations between GEBV were observed for HSD, Max_Tv_, HSU_A_, HSU_B_, and Nor_medvar, which indicate that these five CR indicators share similar underlying genetic mechanisms. Furthermore, individuals with a higher CR are more likely to exhibit better physiological responses, a higher body condition score, and enhanced reproductive performance under hot conditions. The findings of this study highlight the feasibility of using repeated records for deriving novel indicators of climate adaptation and the potential benefits of genetically selecting more heat-tolerant individuals based on the derived CR indicators.

### Supplementary Information

Additional file 1: Table S1. Spearman rank correlations based on the GEBV between Skew(Med), HSU_A_, HSU_B_, Nor_medvar, HSD, and Max_Tv_ indicators derived from vaginal temperatures in lactating sows. Table S1 contains two sheets (a) ranking list of the top 100 individuals with the highest GEBV and (b) ranking list of the bottom 100 individuals with the highest GEBV.

## Data Availability

All the data supporting the results of this study are included in the article and its Additional file.
